# Bioactivity of Cereal- and Legume-Based Macaroni Pasta Volatiles to Adult *Sitophilus granarius* (L.)

**DOI:** 10.3390/insects12090765

**Published:** 2021-08-26

**Authors:** Pasquale Trematerra, Onofrio Marco Pistillo, Giacinto Salvatore Germinara, Marco Colacci

**Affiliations:** 1 Department of Agricultural, Environmental and Food Sciences, University of Molise, Via de Sanctis, I-86100 Campobasso, Italy; trema@unimol.it; 2 Department of Agricultural Sciences, Food, Natural Resources and Engineering, University of Foggia, Via Napoli 25, I-71122 Foggia, Italy; marco.pistillo@unifg.it

**Keywords:** granary weevil, special pasta, food preferences, HS-SPME/GC-MS

## Abstract

**Simple Summary:**

Pasta factories can be infested by insects. By following the odour of cereal-based pasta, insects can enter packages of commercial products. The aim of this work was to compare the bioactivity of volatiles produced by cereal- and legume-based macaroni pasta on adults of granary weevil, *Sitophilus granarius*, in multi-choice behavioural bioassays. Tests were performed with ten commercially available Italian macaroni pastas made from six different cereals or four different legumes. Granary weevil adults were more attracted to cereal-based pastas than legume-based pastas, but the differences in attractiveness were not always significant. Gas chromatography-mass spectrometry analysis of head-space solid-phase microextraction collections from the different pasta samples highlighted marked qualitative and quantitative differences, with aliphatic aldehydes and aliphatic alcohols being the most abundant volatile components of cereal- and legume-pastas, respectively. Moreover, the results of the two-choice behavioural bioassays suggested that the low level of attraction to legume pasta is mainly due to the lack of attractant stimuli other than emission of repellent compounds.

**Abstract:**

The attractiveness of ten commercially available Italian macaroni pastas made from different cereals [*Triticum durum*; *Triticum durum* (whole wheat); *Triticum dicoccum*; mixture of five cereals; *Triticum turgidum*; *Triticum turanicum*] or legumes (*Cicer arietinum*; *Lens culinaris*; *Pisum sativum*; *Vicia faba*) to *Sitophilus granarius*, was compared. *S. granarius* adults were more attracted to cereal pastas than legume pastas, but the differences in attractiveness were not always significant. Consistent with the results of behavioural bioassays, the mortality of adults over 20 days exposed to pasta samples was 100% with the legume pasta samples and only 8% with the *T. turanicum* pasta. GC-MS analysis of HS-SPME extracts from the different pasta samples highlighted marked qualitative and quantitative differences, with aliphatic aldehydes and aliphatic alcohols being the most abundant volatile components of cereal- and legume-pastas, respectively. In two-choice behavioural bioassays, insect attraction to a 1:1 combination of *T. turanicum* and *C. arietinum* pastas (80%) was even higher than that observed in *T. turanicum* pasta alone (64%) and in *C. arietinum* pasta alone (20%). This strongly suggested that the low attractiveness of legume pasta is mainly due to the lack of attractant stimuli rather than emission of repellent compounds.

## 1. Introduction

Pasta factories can be infested by insects, mainly larvae of *Plodia interpunctella* (Hübner) (Lepidoptera: Pyralidae) and adults of *Lasioderma serricorne* (F.) (Coleoptera: Anobiidae), *Rhyzopertha dominica* (Fabricius) (Coleoptera: Bostrichidae), *Sitophilus* spp. (Coleoptera: Curculionidae) and *Tribolium* spp. (Coleoptera: Tenebrionidae), leading to negative economic and commercial consequences [1,2,3,4,5,6,7,8].

In particular, dried cereal pasta can be infested with *Sitophilus* spp. during shipment in trucks, railcars and ships, as well as during retail storage, or even in the consumer’s home [6,9,10,11,12]. Following the odour of cereal-based pasta, adult weevils (as penetrator) can enter packages of commercial products enlarging the air vent micro-holes, through the openings made by them, or through the existing openings created by poor seals or mechanical damage [6,11,12,13,14,15].

Paper and cardboard packaging are the most commonly used materials, and are generally considered the most susceptible to insect attack [15]. To prevent infestation, resistant and sealed packages can be used [8,16,17,18,19,20]. Most food products available on the market, including cereal-based pasta, are packaged to prevent infestation [20,21,22,23,24], although contamination by insects is still frequent.

In recent years, consumers are requiring new types of pasta with healthy nutritional characteristics, as a good source of plant-based protein and fibre, certified gluten free and non-GMO. For these reasons, the food industry has produced and marketed pasta made with flours derived from legumes. The legume-based pasta production process is very similar to that of wheat-based pasta. The dried legumes are milled and then run through fine-mesh sieves until only a fine-textured flour remains. Water is mixed into the flour, other ingredients are sometimes added, and then the dough is kneaded and finally extruded through dies into various shapes.

To our knowledge, there is no published scientific literature that considers *Sitophilus granarius* (L.) infestations in dried pasta made with legume flour. However, there are some studies that consider the relationship between *S. granarius* adults and legume seeds [25,26].

The granary weevil, *S. granarius*, is distributed throughout the temperate regions of the world and is a frequent pest of wheat, also attacking barley, maize, sorghum, rice, and other cereal grains. Larval stages feed inside the kernels, leaving only the hulls. They sometimes infest sunflower seeds, dried beans, chickpeas, peanuts, fava beans, acorns, chestnuts, pasta products, and ornamental dried corn [9,27].

It is known that legume seeds contain a wide range of allelochemicals with toxic and deterrent effects against insect pests [28,29]. An admixture of yellow split-peas (*Pisum sativum* L.) with wheat resulted in a marked reduction in the survival and reproduction rate of *Sitophilus oryzae* (L.) [30,31]. 

Concentrations as low as 0.01% pea protein were shown to cause adult mortality and reduced reproduction for several stored-product insect pests [32,33]. The repellence of pea seed fractions to stored-product insect pests has been demonstrated in multiple-choice tests, in which wheat kernels were dusted with fractions rich in either protein, fibre or starch. This result is probably due to either the olfactory or gustatory effects of the pea fractions [25,26]. However, to the best of our knowledge, semiochemical interactions between stored-product pests and legume-derived products remain little investigated. 

The main purpose of this work was to compare the bioactivity of volatiles produced by cereal- and legume-based macaroni pasta against adults of *S. granarius* in multi-choice behavioural bioassays. Moreover, head-space solid-phase microextraction (HS-SPME) extracts from the different types of pasta tested in bioassays were analysed by gas-chromatography coupled with mass-spectrometry (GC-MS) to highlight differences between their odour blends.

## 2. Materials and Methods

### 2.1. Insects

A wild *S. granarius* population found and reared on barley with no history of exposure to insecticides was maintained in a climatic chamber at 28 ± 2 °C and 70 ± 5% RH, with an L12:D12 photoperiod. Unsexed 1 to 2-week-old adults were used in behavioural bioassays and susceptibility tests.

### 2.2. Pasta Materials

Ten commercially-available Italian macaroni pasta brands of different types and shapes (penne and rigatoni) (samples A–J) made from cereal-based (*Triticum durum* Desf. (A); *Triticum durum* (whole wheat) (B); *Triticum dicoccum* L. (C); five cereals (wheat, spelt, barley, maize and rye) (D); *Triticum turgidum* L., Kamut (E); and *Triticum turanicum* Jakubz., Khorasan (F), or legume-based (*Lens culinaris* Medik (G); *Cicer arietinum* L. (H); *Pisum sativum* L. (I); and *Vicia faba* L. (J)) flours were used. 

The nutritional information (average values) for 100 g of each macaroni pasta is reported in Table 1.

### 2.3. Multiple-Choice Behavioural Bioassays

The attractiveness of different macaroni pastas to adult granary weevil adults was compared using a circular olfactometer arena (100 cm diameter, 50 cm height) similar to that described in previous studies [5]. 

Modified Petri dishes (9 cm diameter), each baited with a sample (25 g) of different macaroni pastas, were equally spaced along the edge of the arena, and 100 *S. granarius* adults were released at its centre. To prevent insect escape, Teflon paint was applied to the arena walls. The number of “trapped” insects was counted 1 day and 7 days after their introduction to the arena. After each experiment, baits were renewed and the positions of the different Petri dishes were randomly assigned. Tests were carried out in the dark at 28 ± 2 °C and 70 ± 5% RH. For each experiment, six replicates were performed.

### 2.4. Two-Choice Behavioural Bioassays

The attractiveness of Khorasan pasta (F) and chickpea pasta (H) was compared using the circular olfactometer arena, the modified Petri dishes and the methodology described above. The numbers of trapped and untrapped (free in the arena) insects were checked 1 day after their introduction to the arena. The following pairwise comparisons were performed (First vs. Second choice): 25 g of *Triticum turanicum* vs. control (empty modified Petri dish); 25 g of *Cicer arietinum* vs. control; mixed 25 g of *Triticum turanicum* and 25 g of *Cicer arietinum* vs. control; mixed 50% *Triticum turanicum* and 50% *Cicer arietinum* vs. mixed 75% *Triticum turanicum* and 25% *Cicer arietinum*; mixed 50% *Triticum turanicum* and 50% *Cicer arietinum* vs. mixed 25% *Triticum turanicum* and 75% *Cicer arietinum*; and 100% *Triticum turanicum* vs. 100% *Cicer arietinum*. For each experiment, four replicates were performed (see Table 2).

In each trial, a response index (RI) was calculated using RI = [(T − C)/Tot] × 100, where T is the number of insects responding to the first choice, C is the number responding to the second choice, and Tot is the total number of insects released [13].

### 2.5. Susceptibility of Pasta Samples

For each type of pasta tested, 25 g samples were placed in wide-necked vials (100 mL volume) and infested with 30 unsexed 1 to 2-week-old granary weevil adults. To ensure air exchange, a series of small holes were punched in the vial screw caps. Vials were maintained in the dark at 28 ± 2 °C and 70 ± 5% RH. Granary weevil mortality was recorded 20 days after the start of the experiment, and emergence of new adults (F1) was checked every 2 days for 8 consecutive weeks. For each experiment, four replicates were performed.

### 2.6. Extraction of Pasta Volatiles

To identify and quantify the volatile compounds emitted by the different types of pasta tested in the behavioural bioassays, the static head-space solid-phase micro-extraction (HS-SPME) technique was used according to Beleggia et al. (2009) and Germinara et al. (2019) [34,35]. Pastas were stored in their unopened packages at room temperature until use. Before analysis, 10 g of each pasta sample was ground in an electric mill for 20 s at 6000 rpm (Waring^®^ laboratory blenders, Fisher scientific, Göteborg, Sweden) and placed in a 20 mL headspace vial (Supelco Co., Bellefonte, PA, USA), sealed with a PTFE/silicon septum (Supelco Co., Bellefonte, PA, USA) for analysis. The vial was then conditioned at 50 °C for 30 min in a water bath prior to SPME headspace sampling.

Extraction was performed using SPME fibres (Supelco Co., Bellefonte, PA, USA) coated with either 50/30 μm of divinylbenzene–carboxen–polydimethylsiloxane (DVB–CAR–PDMS). The fibres were conditioned before use by heating them in the injection port of the GC system, according to the manufacturer’s recommendations, at 270 °C for 1 h. Then, the SPME needle was introduced through the septum, and the fibre was exposed in the vial to the headspace of the pasta sample for 90 min. A temperature of 50 °C was maintained during headspace sampling. After the extraction time, the fibre was recovered and transferred to the injection port of the GC, where the compounds were thermally desorbed at 250 °C for 4 min. A fibre cleaning step of 10 min at the conditioning temperature with the split valve opened was performed in the GC injector after every chromatographic run to remove any absorbed residue. Before the acquisitions, a blank test was performed under the same experimental conditions to check for possible impurities. Each sampling was performed in triplicate.

### 2.7. Gas Chromatography-Mass Spectrometry (GC-MS)

GC–MS analyses were performed using an Agilent 7890B series gas chromatograph (Agilent Technologies, Milan, Italy) coupled with an Agilent 5977A mass selective detector (MSD) equipped with an HP-5MS capillary column (30 m × 0.25 mm ID, 0.5 µm film thickness, J&W Scientific Inc., Folsom, CA, USA). The desorption step was carried out in the splitless mode (4 min) with a programmed temperature from 60 °C to 250 °C at 5 °C/min, with a final holding time of 15 min. Spectra were recorded in the electron impact mode (ionization energy, 70 eV) in a range of 15–550 amu at 2.9 scans/s. The identification of volatile compounds was achieved by comparing mass spectra with those of the data system library (NIST08, *p* > 90%), and, wherever possible, by comparing retention times (R.T.) and mass spectra with those of commercially available standards. Moreover, a mixture of a continuous series of straight-chain hydrocarbons, C5–C40 (Alkane Standard Solution C6–C40, Sigma Aldrich, Milan, Italy), was injected into an HP-5MS column under the same conditions previously described for the pasta samples to obtain the linear retention indices (RIs) [36]. Component relative percentages were calculated based on GC peak areas. Each extract was analysed in triplicate.

### 2.8. Data Analysis

For the multiple-choice behavioural bioassays, the numbers of insects found in the different dishes were subjected to Friedman two-way ANOVA by ranks. In the case of significance (*p* < 0.05), the Wilcoxon signed ranks test was used for separation of means.

For the two-choice behavioural bioassays, Student’s *t*-test was used to compare the mean numbers of insects found in the two choices.

The data on susceptibility of pasta samples were submitted to one-way analysis of variance (ANOVA). Means were separated using the Tukey-Kramer honest significant difference (HSD) test at the 0.05 significance level [37].

Statistical analyses were performed using SPSS statistical software 13.0 (SPSS Inc., Chicago, IL, USA).

## 3. Results

### 3.1. Multiple-Choice Behavioural Bioassays

**Figure 1:**
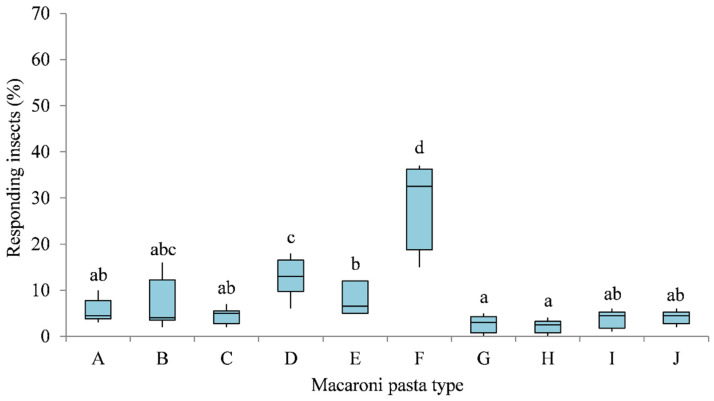
Olfactory responses of *S. granarius* adults to Durum wheat pasta (**A**), Durum whole wheat pasta (**B**), Spelt pasta (**C**), Five cereals pasta (**D**), Kamut pasta (**E**), Khorasan pasta (**F**), Lens pasta (**G**), Chickpea pasta (**H**), Pea pasta (**I**), and Faba bean pasta (**J**) macaroni pasta in 1-day multiple-choice bioassays. Each box plot indicates the median and its range of dispersion (lower and upper quartiles and outliers). Above each box plot, different letters indicate significant differences (Wilcoxon test, *p* < 0.05).

**Figure 2:**
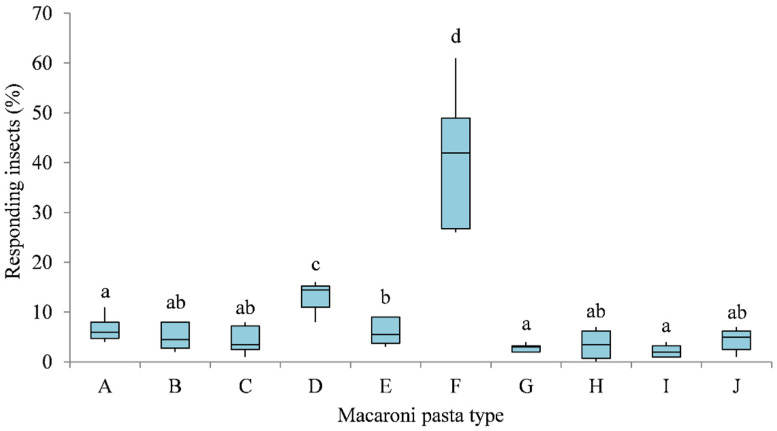
Olfactory responses of *S. granarius* adults to Durum wheat pasta (**A**), Durum whole wheat pasta (**B**), Spelt pasta (**C**), Five cereals pasta (**D**), Kamut pasta (**E**), Khorasan pasta (**F**), Lens pasta (**G**), Chickpea pasta (**H**), Pea pasta (**I**), and Faba bean pasta (**J**) macaroni pasta in 7-days multiple-choice bioassays. Each box plot indicates the median and its range of dispersion (lower and upper quartiles and outliers). Above each box plot, different letters indicate significant differences (Wilcoxon test, *p* < 0.05).

Olfactory responses of granary weevil adults to different pasta samples in multiple-choice behavioural bioassays are reported in Figure 1 and Figure 2. Significant differences in adults captured by the Petri dishes containing different macaroni pastas were recorded 1 day (χ^2^ = 35.029, df = 9, *p* < 0.001) and 7 days (χ^2^ = 35.553, df = 9, *p* < 0.001) after the start of the experiment.

After 1 day, *S. granarius* showed preference, in decreasing order, for Khorasan pasta (28.83%), five cereal pasta (12.83%), Kamut pasta (7.83%), durum whole wheat pasta (6.83%), durum wheat pasta (5.50%), faba bean pasta (4.17%), spelt pasta (4.50%), pea pasta (3.83%), lens pasta (2.67%), and chickpea pasta (2.17%), indicating an overall lower attractiveness for legume-based pastas. The number of insects attracted to the Khorasan pasta was significantly higher (Wilcoxon test, *p* < 0.05) than those attracted to the remaining pasta samples. Lentil and chickpea pastas attracted the lowest numbers of adult insects, which were not significantly different (Wilcoxon test, *p* > 0.05) from the numbers attracted by other legume, spelt, and durum wheat pastas (Figure 1). 

Similar results were obtained after 7 days in the olfactometer arena. *S. granarius* showed preference, in decreasing order, for Khorasan pasta (40.50%), five cereal pasta (13.33%), Kamut pasta (6.00%), durum wheat pasta (6.50%), durum whole wheat pasta (5.00%), faba bean pasta (4.50%), chickpea pasta (3.50%), spelt pasta (4.33%), lens pasta (2.83%), and pea pasta (2.17%) (Figure 2). The number of insects attracted to Khorasan pasta was significantly higher than those attracted to the other pasta samples (Wilcoxon test, *p* < 0.05). The number of insects attracted to five cereal pasta was significantly higher (Wilcoxon test, *p* < 0.05) than those attracted to the durum wheat, spelt and various legume-based pastas. The latter were the weakest attractants, without significant differences among them (Wilcoxon test, *p* > 0.05).

### 3.2. Two-Choice Behavioural Bioassays

The results of the two-choice behavioural bioassays are reported in Table 2. When individually compared with an empty Petri dish, Khorasan, chickpea, and a 1:1 mixture of both pastas attracted 64%, 20%, and 80% of test insects, respectively. In total, 73.5% of insects chose the Khorasan pasta when presented with Khorasan pasta and chickpea pasta, whereas a preferential orientation of adult insects was not observed when making a choice between mixtures of Khorasan and chickpea pastas in different proportions.

### 3.3. Susceptibility of Pasta Samples

The mean numbers of live and dead *S. granarius* adults 20 days after exposure to different pasta samples in wide-necked vials are reported in Table 3. According to our results, significant differences were recorded in weevil mortality (F = 32.869; df = 9, 30; *p* < 0.01). A 100% mortality rate of adult insects was recorded in all legume-based pastas, significantly higher than those recorded for durum wheat pasta (70.00%), five cereal pasta (68.33%), and Khorasan pasta (27.50%).

After the incubation period, new adults (F1 progeny) emerged from the Khorasan pasta (ten samples) and from the durum whole wheat pasta (one sample). No adults emerged from the other types of macaroni pasta.

### 3.4. Characterisation of Pasta Volatiles

HS-SPME/GC-MS was used to detect the main components in the odour profile of the different pasta samples. The percentages of specific compounds, expressed as relative abundance, are reported in Table 4. Across all ten pastas, a total of 50 volatile compounds in the chemical classes of alcohols, aldehydes, ketones, esters, lactones, terpenes, hydrocarbons, furans, and other compounds were detected.

A total of 27, 34, 40, 34, 33, and 33 volatile compounds were respectively identified in the head-space of samples A, B, C, D, E, and F, obtained from different cereals, whereas 42, 43, 30, and 40 compounds were identified from the G, H, I, and J legume-based pasta samples.

In cereal-based pasta samples, aldehydes were the most represented chemical class, both in terms of the number of compounds (10–13) and relative abundance (40.14–61.76%), followed by alcohols, aromatics, furans, and hydrocarbons. In the head-space volatile fraction of legume-based pasta samples, alcohols were the most numerous (11–12) and abundant (53.8–61.15) among different chemical classes, followed by aldehydes, aromatics, furans, and lactones.

In the head-space fraction of all cereal-based pasta samples, hexanal was the most abundant compound (23.24–38.66%). In these samples, further major volatile components were nonanal (11.64%) and benzaldehyde (9.24%) in sample A (wheat flour); 2-pentylfuran (12.20%) and tridecane (7.37%) in sample B (wholemeal wheat flour); 2-pentylfuran (22.0%) and tridecane (10.4%) in sample C (wholemeal spelled flour); pentylfuran (9.20%), nonanal (6.22%), and benzaldehyde (6.04%) in sample D (five cereal flour); nonanal (8.87%) and benzaldehyde (7.78%) in sample E (Kamut flour); and 2-pentylfuran (6.10%) and benzaldehyde (4.62%) in sample F (Khorasan flour). Samples E and F emitted similar substances, with the exception of the total aldehydes, which were present in a higher percentage in sample E, and a higher percentage of alcohols in sample F.

Hexenol was the main volatile compound identified in the head-space fraction of all legume-based pastas. Additional major volatile components of these samples were hexanal (8.33%), 2-pentylfuran (7.41%), and 1-octen-3-ol (6.81%) in sample G (lentil flour); 2-pentylfuran (9.49%), hexanal (6.00%), styrene (4.40%), and gamma-lactone (4.23%) in sample H (chickpea flour); hexanal (10.03%), 1-octen-3-ol (5.19%), and 1-pentanol (4.24%) in sample I (pea flour); and styrene (9.51%), hexanal (8.59%), and 1-octen-3-ol (5.24%) in sample J (fava bean flour).

## 4. Discussion

The results of multiple-choice behavioural bioassays showed that *S. granarius* adults are able to selectively respond to odours of different types of Italian cereal- and legume-based pastas. In fact, in both the day one and day seven tests, cereal-based pastas were overall more attractive than legume-based ones. Among all pasta samples tested, those made from *T. turanicum* (Khorasan) and five cereals (wheat, spelt, barley, maize, and rye) were the most attractive. Whereas, those from whole wheat *T. durum*, *T. dicoccum*, and particularly from different legumes, were the weakest attractants.

To investigate the nature of the low attractiveness of legume-based pastas to granary weevil adults, two-choice behavioural bioassays were performed. In these experiments, pastas obtained from *T. turanicum* (Khorasan pasta) and *C. arietinum* (chickpea pasta) confirmed their respective high and low attractiveness to adult insects. Whereas, a 1:1 mixture of the two pastas elicited a significant insect attraction, comparable to that of *T. turanicum* pasta. This strongly suggests that the low attractiveness of legume-based pastas to granary weevils mainly depends on the lack of attractive odour stimuli rather than the presence of repellent volatile compounds. This hypothesis was further confirmed by pairwise comparisons of different mixtures of the two types of pasta where a preferential insect orientation was not observed due to the presence of both the attractive (Khorasan pasta) and the non-attractive source (chickpea pasta) in both test odour-stimuli.

Several studies have highlighted the importance of ratios and concentrations of volatiles for host location by phytophagous insects [38,39,40,41,42]. Male and female granary weevil antennae are capable of selectively perceiving a wide range of individual cereal grain volatiles [43] and kernel solvent extracts from different durum and bread wheat genotypes [35]. In behavioural bioassays testing various concentrations of 20 EAG (Electroantennography)-active volatiles, five compounds (1-butanol, 3-methyl-1-butanol, pentanal, maltol, and vanillin) acted uniquely as attractants, three compounds (1-pentanol, (*E*,*E*)-2,4-heptadienal, and phenylacetaldehyde) were attractive at lower concentrations but repellent at higher doses, and 12 compounds (1-hexanol, butanal, hexanal, heptanal, (*E*)-2-hexenal, (*E*,*E*)-2,4-nonadienal, (*E*,*E*)-2,4-decadienal, 2,3-butanedione, 2-pentanone, 2-hexanone, 2-heptanone, and furfural) were repellent at high doses, strongly suggesting that host finding behaviour of granary weevils depends on the balance of positive and negative semiochemical stimuli [13]. 

In the head-space of different pasta samples, a total of 50 volatile compounds were identified by chemical analysis. Even though many of these compounds were found in the head-space fractions of both cereal- and legume-based pastas, differences in their relative proportions along with qualitative differences were highlighted. In fact, the odour profiles of cereal-based pastas mainly contained aldehydes, followed by alcohols, aromatics, furans, and hydrocarbons whereas those of legume-based pastas were characterised by a high content of alcohols, followed by aldehydes, aromatics, furans, and lactones. Short chain aldehydes are lipid oxidation products of the hydroperoxide lyase pathway of oxylipin metabolism [44,45], which can be converted into the corresponding alcohols by the action of alcohol dehydrogenase [46]. Ketones and hydrocarbons are also derived from lipid oxidation, from both enzymatic and non-enzymatic oxidative degradations, while terpenes are naturally present/synthesised by the plant [47]. Lactones are strongly related to legumes [48]. Furans are mainly produced by the Maillard reaction during pasta drying [34,49]. Differences in composition of the pasta volatile fractions might account for differences in attractiveness of various pasta samples to granary weevil adults.

In our study, *S. granarius* adults strongly preferred Khorasan and, to a lesser extent, five cereal pastas over the other samples tested, with legume-based pastas being less preferred. However, by matching the volatile profiles of pasta samples with the known behavioural activity of some of their components, it is difficult to draw conclusions about the compounds involved in determining the olfactory preference of *S. granarius* adults. In fact, the volatile fractions of both cereal- and legume-based pastas are comprised of some of the above-mentioned granary weevil attractants and repellents [13], but they represent only a limited number of VOCs, identified in this study, and the behavioural activity of many of these components is still unknown [50]. Therefore, the contributions of other, even minor, volatile compounds emitted by the most attractive pasta samples, alone and in combination with known attractants, deserve further investigation.

The response pattern of insects to different pasta samples did not vary markedly between 1 and 7 days; this strongly suggests that the olfactory preference of insects was mainly determined by the first choice made in response to the odour profiles of different pasta samples. However, since insects remained alive and fed on the pastas during the experiments, the possible release of additional volatiles, mainly by insects, which were not detected in the SPME collections from the pasta samples alone, cannot be excluded. Further studies are needed to confirm differences in the attractiveness of pasta samples to granary weevils in short-term behavioural bioassays and to characterize the SMPE volatile profiles of pastas fed upon by insects to better understand the chemical bases of attraction.

As reported, in susceptibility tests, 100% mortality of adult weevils was recorded 20 days after their exposure to all legume-based pastas and no progeny were recorded during the eight consecutive weeks. Therefore, consistently with results of behavioural bioassays, susceptibility tests demonstrated the unsuitability of legume-based pastas as a food source for adult granary weevils. This is in fairly good agreement with previous studies reporting a significant reduction of survival and reproduction of conspecific *S. oryzae* adults fed with an admixture of yellow split-peas and wheat [30,31] or rice treated with 1% pea flour extract [51]. Similar results were obtained by Fields et al. (2001) [25] with nine stored-grain beetles, including *S. oryzae*, *S. granarius*, and *S. zeamais*, reared on wheat kernels or flour treated with *P. sativum* fractions. Altogether, *Sitophilus* spp. were the most sensitive species and the protein-rich pea fraction was more toxic than the fibre fraction, which was more toxic than the starch fraction [26]. 

The toxicity of pea albumin 1b (PA1b), a 37 amino-acid peptide extracted from pea seeds, for cereal weevils (*Sitophilus* spp.) was discovered by Delobe et al. (1998) [33], and a high-affinity binding site for this entomotoxin in susceptible *Sitophilus* strains was characterised [52]. However, the diverse biological activities of legume fractions towards stored-product pests strongly suggest that the contents of different toxic and deterrent allelochemicals in legume flour remain active, even after the pasta production process, as demonstrated by our study. 

## 5. Conclusions

From a practical perspective, it is interesting to define the biological activity of volatile and non-volatile components of legume-based pastas, as they could allow the identification of possible repellent, deterrent, and toxic compounds to be used, for example, for the preparation of bioactive packaging able to limit the risk of infestation of packaged products [21,50]. However, more work is needed to determine the linkage between the bioassays and the volatile compounds from the chromatography studies.

## Figures and Tables

**Table 1:** Nutrition facts of the ten different types of commercial Italian macaroni pasta used in the tests.

	Samples	Pasta Type	Cereal/Legume	Energy Value	Fats g/100 g	Carboydrates g/100 g	Fibers g/100 g	Proteins g/100 g	Salt g/100 g
Cereal pasta	A	Durum wheat pasta	*Triticum durum*	359 kcal 1525 kj	1 0.3 satur.	74.0 3.5 sugars	3.0	13.5	0.03
	B	Durum whole wheat pasta	*Triticum durum*	347 kcal 1468 kj	2.20 0.5 satur.	64.5 2.5 sugars	6.5	14.0	0.004
	C	Spelt pasta	*Triticum dicoccum*	351 kcal 1485 kj	3.1 0.5 g satur.	65.0 sugars 3.2	7.0	12.0	0.06
	D	Five cereals pasta	wheat, spelt, barley, maize, rye	352 kcal 1484 kj	2.2 0.5 satur.	67.0 sugars 3.0	6.8	12.5	0.007
	E	Kamut pasta	*Triticum turgidum*	350 kcal 1483 kj	1.3 0.3 satur.	69.0 sugars 4.3	3.0	14.0	0.00
	F	Khorasan pasta	*Triticum turanicum*	346 kcal 1453 kj	1.4 0.3 satur.	68.0 sugars 2.5	4.8	13.0	0.03
Legume pasta	G	Lens pasta	*Lens culinaris*	325 kcal 1375 kj	0.3 0.2 satur.	48.0 sugars 1.2	13.6	25.8	0.05
	H	Chickpea pasta	*Cicer arietinum*	345 kcal 1457 kj	3.1 1.6 satur.	53.8 sugars 1.8	11.1	20.0	0.06
	I	Pea pasta	*Pisum sativum*	334 kcal 1415 kj	0.5 0.3 satur.	57.5 sugars 7.2	6.7	21.4	0.05
	J	Faba bean pasta	*Vicia faba*	334 kcal 1412 kj	0.5 0.3 satur.	54.3 sugars 2.6	6.3	24.7	0.05

**Table 2:** Behavioural responses of *S. granarius* adults in two-choice bioassay. In a row, significant differences between first and second choices are indicated by Student’s *t*-test.

Two-Choice Bioassay	First Choice (±SE)	Second Choice (±SE)	Student’s *t*-Test		Response Index (±SE)
First vs. Second Choice			*t*-Value	*p*-Value	
F vs. Control	64.50 ± 2.25	1.25 ± 0.25	<0.001	28.111	63.25 ± 2.25
H vs. Control	20.00 ± 1.08	3.50 ± 0.65	0.001	13.863	16.50 ± 1.19
F+H (1:1) vs. Control	80.00 ± 1.47	2.00 ± 0.41	<0.001	63.687	78.00 ± 1.22
F vs. H	73.75 ± 1.55	4.75 ± 0.48	<0.001	35.242	69.00 ± 1.96
F+H (1:1) vs. F+H (3:1)	40.75 ± 2.84	39.25 ± 4.33	0.828	0.236	1.50 ± 6.34
F+H (1:1) vs. F+H (1:3)	39.00 ± 1.78	41.50 ± 1.32	0.464	–0.837	−2.5 ± 2.99

**Table 3:** Percentage of dead adults (±SE) checked after 20 days of contact with the macaroni pasta samples and number of progeny (F1) emergence.

Samples	Pasta	Percentage of Dead Adults (±SE) *	Number of Progeny Emergence
A	Durum wheat pasta	70.00 ± 5.27 ^b^	0
B	Durum whole wheat pasta	76.67 ± 3.60 ^bc^	1
C	Spelt pasta	85.83 ± 2.50 ^bc^	0
D	Five cereals pasta (wheat, spelt, barley, maize rye)	68.33 ± 5.00 ^b^	0
E	Kamut pasta	73.33 ± 4.30 ^bc^	0
F	Khorasan pasta	27.50 ± 4.97 ^a^	10
G	Lens pasta	100.00 ^c^	0
H	Chickpea pasta	100.00 ^c^	0
I	Pea pasta	100.00 ^c^	0
J	Faba bean pasta	100.00 ^c^	0

* Means followed by the same letter are not significantly different (Tukey-Kramer HSD test at *p* < 0.05).

**Table 4:** VOCs levels detected in the head-space of different cereal and legume pasta samples.

					Area (%) ± S.E. ^1^									
Peak No.	Compound	R.T	RI_cal_^2^	RI_ref_^3^	A	B	C	D	E	F	G	H	I	J
	Aldehydes													
3	3-Methylbutanal	1.70	665	668	0.69 ± 0.01	-	2.11 ± 0.03	0.74 ± 0.01	-	-	-	-	-	-
4	2-Methylbutanal	1.75	669	668	-	1.35 ± 0.02	1.08 ± 0.01	0.72 ± 0.01	-	-	-	-	-	-
5	Pentanal	1.91	709	706	1.07 ± 0.03	0.66 ± 0.03	1.61 ± 0.03	1.89 ± 0.04	1.43 ± 0.02	1.82 ± 0.07	0.37 ± 0.03	0.22 ± 0.02	0.51 ± 0.01	-
8	Hexanal	2.85	803	801	33.31 ± 0.64	32.66 ± 0.36	23.24 ± 0.07	35.4 ± 0.26	38.01 ± 0.08	38.66 ± 0.36	8.33 ± 0.10	6.00 ± 0.07	10.03 ± 0.08	8.59 ± 0.21
13	Heptanal	4.92	901	902	2.81 ± 0.03	-	1.14 ± 0.02	2.24 ± 0.05	1.05 ± 0.04	1.44 ± 0.07	-	0.13 ± 0.01	0.58 ± 0.06	-
15	(*E*)-2-Heptenal	6.45	957	954	3.32 ± 0.05	-	2.14 ± 0.02	2.47 ± 0.07	3.2 ± 0.09	1.82 ± 0.04	0.91 ± 0.01	0.54 ± 0.03	1.15 ± 0.03	0.65 ± 0.02
23	Octanal	7.87	996	998	1.53 ± 0.01	0.82 ± 0.01	0.66 ± 0.01	0.87 ± 0.02	1.13 ± 0.07	0.76 ± 0.06	0.41 ± 0.01	1.06 ± 0.03	1.24 ± 0.05	-
30	(*E*)-2-Octenal	9.58	1051	1054	1.42 ± 0.02	1.08 ± 0.03	2.93 ± 0.04	1.18 ± 0.05	1.18 ± 0.08	1.36 ± 0.10	1.65 ± 0.04	1.58 ± 0.03	0.41 ± 0.01	1.66 ± 0.04
34	Nonanal	11.00	1096	1100	11.64 ± 0.38	3.09 ± 0.05	2.15 ± 0.02	6.22 ± 0.07	8.87 ± 0.18	3.48 ± 0.02	1.26 ± 0.03	2.54 ± 0.12	3.70 ± 0.10	1.93 ± 0.03
36	(*E*)-2-Nonenal	12.75	1160	1157	-	1.82 ± 0.03	1.22 ± 0.06	2.77 ± 0.05	3.13 ± 0.07	1.12 ± 0.07	0.63 ± 0.02	0.69 ± 0.01	0.89 ± 0.02	0.88 ± 0.03
39	Decanal	14.07	1202	1201	2.83 ± 0.01	0.98 ± 0.07	0.71 ± 0.01	1.86 ± 0.04	2.81 ± 0.12	1.04 ± 0.10	0.27 ± 0.03	0.67 ± 0.03	0.59 ± 0.02	0.71 ± 0.01
40	2,4 Nonadienal	14.30	1215	1217	-	0.48 ± 0.01	0.34 ± 0.01	0.16 ± 0.01	0.22 ± 0.01	0.18 ± 0.05	0.07 ± 0.01	0.16 ± 0.02	-	-
46	2-Butyl-2-octenal	18.72	1353	1360	0.47 ± 0.01	0.91 ± 0.02	0.81 ± 0.06	0.64 ± 0.02	0.73 ± 0.02	2.50 ± 0.09	-	0.21 ± 0.01	-	-
	Total aldehydes				59.09 ± 1.15	43.85 ± 0.58	40.14 ± 0.69	57.16 ± 0.62	61.76 ± 0.71	54.18 ± 1.01	13.9 ± 0.25	13.8 ± 0.31	19.1 ± 0.35	14.42 ± 0.30
	Alcohols													
6	3-Methylbutanol	2.15	638	740	-	-	0.25 ± 0.02	-	-	-	-	0.15 ± 0.03	0.53 ± 0.03	0.60 ± 0.02
7	1-Pentanol	2.44	765	771	2.02 ± 0.08	1.95 ± 0.02	2.15 ± 0.03	2.47 ± 0.05	3.07 ± 0.04	4.85 ± 0.05	3.28 ± 0.11	4.08 ± 0.04	4.24 ± 0.07	2.43 ± 0.11
9	1-Hexanol	4.11	867	870	1.89 ± 0.02	3.3 ± 0.05	3.92 ± 0.04	1.25 ± 0.05	1.99 ± 0.07	2.57 ± 0.21	42.09 ± 0.51	38.22 ± 0.24	45.06 ± 0.24	39.97 ± 0.22
12	2-Heptanol	4.87	891	896	2.23 ± 0.05	1.53 ± 0.02	0.76 ± 0.01	-	0.71 ± 0.29	0.78 ± 0.02	2.27 ± 0.08	0.86 ± 0.03	1.83 ± 0.03	0.86 ± 0.03
17	1-Heptanol	6.86	961	966	-	0.18 ± 0.01	0.36 ± 0.01	-	-	0.73 ± 0.04	1.93 ± 0.03	3.00 ± 0.07	2.30 ± 0.10	0.75 ± 0.02
18	1-Octen-3-ol	7.15	969	971	3.81 ± 0.04	2.39 ± 0.02	3.48 ± 0.02	4.13 ± 0.07	3.42 ± 0.09	3.71 ± 0.02	6.81 ± 0.01	2.47 ± 0.14	5.19 ± 0.10	5.24 ± 0.05
21	3-Octanol	7.65	987	991	-	-	-	-	-	-	0.52 ± 0.02	-	0.62 ± 0.02	0.25 ± 0.02
22	2-Octanol	7.80	984	994	1.83 ± 0.03	0.66 ± 0.02	0.52 ± 0.01	0.86 ± 0.02	1.43 ± 0.06	1.45 ± 0.03	1.15 ± 0.06	0.70 ± 0.02	0.87 ± 0.03	0.69 ± 0.02
26	2-Ethylhexanol	8.68	1036	1038	0.62 ± 0.01	0.75 ± 0.02	1.21 ± 0.05	-	-	-	-	0.12 ± 0.00	-	0.59 ± 0.02
31	(*E*)-2-Octenol	9.91	1057	1060	0.22 ± 0.01	-	-	0.31 ± 0.01	-	-	0.56 ± 0.03	0.55 ± 0.03	0.93 ± 0.03	0.42 ± 0.02
32	1-Octanol	9.99	1059	1063	-	0.42 ± 0.01	-	0.43 ± 0.02	0.65 ± 0.03	0.94 ± 0.03	1.33 ± 0.02	3.86 ± 0.05	2.82 ± 0.08	0.68 ± 0.03
33	2,5-Dimethylcyclohexanol	10.68	1084	1099	-	-	-	-	0.52 ± 0.06	2.03 ± 0.08	0.23 ± 0.02	0.85 ± 0.03	0.23 ± 0.01	-
37	1-Nonanol	13.05	1158	1165	-	-	-	-	-	-	0.98 ± 0.07	2.28 ± 0.09	1.21 ± 0.05	1.00 ± 0.06
	Total alcohols				12.62 ± 0.19	11.18 ± 0.15	12.65 ± 0.16	9.45 ± 0.20	11.79 ± 0.58	17.06 ± 0.41	61.15 ± 0.93	57.14 ± 0.72	65.83 ± 0.77	53.48 ± 0.58
	**Ketones**													
1	2-Propanone	1.33	481	487	-	2.43 ± 0.07	0.76 ± 0.01	1.42 ± 0.02	1.72 ± 0.01	0.52 ± 0.02	0.26 ± 0.03	-	-	0.48 ± 0.01
10	2-Heptanone	4.63	881	889	-	-	2.09 ± 0.05	0.92 ± 0.03	-	-	-	-	0.49 ± 0.02	-
28	3-Octen-2-one	8.99	1027	1030	-	0.15 ± 0.01	0.42 ± 0.01	-	0.33 ± 0.01	1.77 ± 0.07	0.55 ± 0.03	1.53 ± 0.67	1.08 ± 0.08	0.41 ± 0.30
	Total ketones				0	2.58 ± 0.05	3.27 ± 0.04	2.34 ± 0.04	2.05 ± 0.01	2.29 ± 0.07	0.81 ± 0.05	1.53 ± 0.67	1.57 ± 0.06	0.89 ± 0.26
	Terpenes													
14	α-Pinene	5.80	931	939	-	0.41 ± 0.01	0.23 ± 0.01	0.54 ± 0.03	-	-	0.27 ± 0.01	0.20 ± 0.01	0.50 ± 0.03	1.69 ± 0.03
19	Sulcatone	7.38	973	974	2.20 ± 0.06	2.08 ± 0.04	1.39 ± 0.01	1.53 ± 0.02	0.54 ± 0.06	0.80 ± 0.04	0.63 ± 0.03	-	0.21 ± 0.01	0.34 ± 0.01
25	Limonene	8.66	1028	1031	-	0.95 ± 0.02	0.34 ± 0.02	1.09 ± 0.03	0.54 ± 0.06	0.46 ± 0.05	0.43 ± 0.03	0.13 ± 0.01	-	0.72 ± 0.01
49	Geranyl acetone	20.71	1451	1455	0.65 ± 0.02	0.5 ± 0.03	0.52 ± 0.01	0.64 ± 0.02	1.06 ± 0.04	-	0.13 ± 0.01	-	-	0.08 ± 0.01
	*Total terpenes*				2.85 ± 0.05	3.94 ± 0.09	2.48 ± 0.04	3.80 ± 0.08	2.14 ± 0.11	1.26 ± 0.06	1.46 ± 0.06	0.33 ± 0.49	0.71 ± 0.07	2.83 ± 0.29
	Aromatics													
11	Styrene	4.67	874	890	2.86 ± 0.04	3.25 ± 0.06	4.36 ± 0.04	1.17 ± 0.05	1.97 ± 0.07	1.20 ± 0.11	3.4 ± 0.04	4.40 ± 0.12	-	9.51 ± 0.14
16	Benzaldehyde	6.57	958	960	9.24 ± 0.24	3.51 ± 0.05	3.6 ± 0.02	6.04 ± 0.11	7.78 ± 0.07	4.62 ± 0.09	0.24 ± 0.01	0.23 ± 0.03	0.47 ± 0.02	0.49 ± 0.02
27	Benzyl alcohol	8.83	1027	1031	0.41 ± 0.01	-	0.76 ± 0.02	-	0.23 ± 0.02	2.45 ± 0.07	0.55 ± 0.02	0.35 ± 0.02	0.34 ± 0.02	1.20 ± 0.06
35	Phenethyl alcohol	11.29	1112	1116	-	-	-	-	-	-	0.22 ± 0.01	0.41 ± 0.01	0.31 ± 0.01	1.63 ± 0.04
	Total aromatics				12.51 ± 0.22	6.76 ± 0.08	8.72 ± 0.07	7.21 ± 0.13	9.98 ± 0.10	8.27 ± 0.21	4.41 ± 0.07	5.39 ± 0.15	1.12 ± 0.03	12.83 ± 0.21
	Lactones													
29	Gamma-Hexalactone	9.44	1048	1056	-	0.26 ± 0.01	0.42 ± 0.01	-	-	-	0.85 ± 0.03	0.72 ± 0.01	0.64 ± 0.03	0.24 ± 0.02
41	Gamma-octalactone	15.56	1243	1250	-	-	-	-	-	-	0.55 ± 0.01	0.49± 0.04	0.47 ± 0.05	-
45	Gamma-nonalactone	18.44	1358	1361	-	-	-	-	-	-	1.12 ± 0.07	4.23 ± 0.04	2.34 ± 0.13	0.47 ± 0.03
	Total lactones				0	0.26 ± 0.01	0.42 ± 0.01	0.00	0.00	0.00	7.12 ± 0.08	5.44 ± 0.06	3.45 ± 0.15	0.71 ± 0.03
	Furans													
20	2-Pentylfuran	7.52	981	988	3.54 ± 0.08	11.88 ± 0.46	12.20 ± 0.07	9.20 ± 0.13	2.37 ± 0.12	6.10 ± 0.12	7.41 ± 0.08	9.49 ± 0.08	3.27 ± 0.09	1.63 ± 0.05
	Hydrocarbons													
38	Dodecane	13.88	1192	1200	-	1.17 ± 0.01	0.82 ± 0.02	0.48 ± 0.03	0.22 ± 0.02	0.41 ± 0.04	0.24 ± 0.03	0.13 ± 0.01	0.07 ± 0.01	0.53 ± 0.01
42	2,6,11-Trimethyldodecane	16.18	1269	1275	0.74 ± 0.02	1.62 ± 0.02	2.25 ± 0.07	0.81 ± 0.02	0.84 ± 0.03	0.54 ± 0.07	0.99 ± 0.06	0.26 ± 0.02	0.04 ± 0.01	1.85 ± 0.04
44	Tridecane	16.73	1287	1300	1.55 ± 0.02	7.37 ± 0.34	5.68 ± 0.1	2.15 ± 0.07	0.35 ± 0.03	1.04 ± 0.12	0.25 ± 0.03	0.66 ± 0.03	0.20 ± 0.01	0.72 ± 0.01
48	Tetradecane	19.37	1396	1400	-	0.67 ± 0.02	0.62 ± 0.01	0.49 ± 0.04	0.39 ± 0.02	0.36 ± 0.06	0.22 ± 0.02	0.14 ± 0.03	0.21 ± 0.02	0.89 ± 0.04
50	Pentadecene	21.71	1488	1492	-	1.07 ± 0.01	1.12 ± 0.03	1.45 ± 0.04	0.87 ± 0.03	0.76 ± 0.04	0.22 ± 0.01	0.04 ± 0.01	0.06 ± 0.01	0.65 ± 0.02
	Total hydrocarbons				2.29 ± 0.08	11.90 ± 0.69	10.49 ± 0.15	5.38 ± 0.18	2.67 ± 0.11	3.11 ± 0.21	1.92 ± 0.13	1.23 ± 0.08	0.58 ± 0.05	4.64 ± 0.09
	Others													
2	Acetic acid	1.48	623	633	-	-	2.02 ± 0.01	-	-	-	-	0.52 ± 0.01	-	1.43 ± 0.02
24	Hexyl acetate	8.22	1001	1007	-	-	-	-	-	0.78 ± 0.05	0.33 ± 0.03	1.57 ± 0.06	0.44 ± 0.03	0.13 ± 0.01
43	Hexanoic acid, pentyl ester	16.42	1274	1282	0.51 ± 0.01	0.77 ± 0.03	0.62 ± 0.01	0.47 ± 0.03	0.33 ± 0.02	0.47 ± 0.05	0.35 ± 0.02	0.48 ± 0.01	0.17 ± 0.01	0.54 ± 0.03
47	Hexanoic acid, hexyl ester	19.04	1374	1383	0.5 ± 0.01	-	-	-	-	-	0.13 ± 0.01	0.72 ± 0.01	-	-
	Total others				1.01 ± 0.01	0.77 ± 0.03	2.64 ± 0.01	0.47± 0.03	0.33 ± 0.02	1.25 ± 0.07	0.81 ± 0.08	3.29 ± 0.04	0.61 ± 0.05	2.10 ±0.02

^1^ N = 3 replicates; ^2^ RI Lit = Linear retention index from literature; ^3^ RI Exp = Determined linear retention index against mixture of n-alkanes (C_5_–C_40_) on HP-5MS column.

## Data Availability

Data are contained within article.

## References

[1] Süss L., Locatelli D.P. (1999). Activity of *Sitophilus oryzae* (L.), *Rhyzopertha dominica* (F.), *Tribolium confusum* Duval and *Plodia interpunctella* (Hbn.) on alimentary pastas. Tec. Molit..

[2] Riudavets J., Lucas E., Pons M.J. (2002). Insects and mites of stored products in the northeast of Spain. IOBC Bull..

[3] Barros G., Maia A., Rodrigues A., Mexia A. Stored product insect pests in pasta residues in Portugal. Proceedings of the 3rd Meeting of COST Action 842, WG-IV.

[4] Trematerra P. Integrated Management of insects infesting pasta factories in Italy. Proceedings of the 5th Meeting COST Action 842, WG-IV: “Bio-Control of Arthropod Pests in Stored Products”.

[5] Trematerra P. (2009). Preferences of *Sitophilus zeamais* to different types of Italian commercial rice and cereal pasta. Bull. Insectol..

[6] Stejskal V., Kucerova Z., Lukas J. (2004). Evidence and symptoms of pasta infestation by *Sitophilus oryzae* (*Curculionidae*; *Coleoptera*) in the Czech Republic. Plant Prot. Sci..

[7] Trematerra P., Süss L. (2006). Integrated pest management in Italian pasta factories. Proceedings of the 9th International Working Conference of Stored-Product Protection, Brazilian Post-Harvest Association.

[8] Athanassiou C.G., Riudavets J., Kavallieratos G. (2011). Preventing stored-product insect infestations in packaged-food products. Stewart Postharvest Rev..

[9] Longstaff B.C. (1981). Biology of the grain pest species of the genus *Sitophilus* (Coleoptera: Curculionidae): A critical review. Prot. Ecol..

[10] Suss L., Savoldelli S. (2011). Egg mortality of pasta pests during pasta making. Tec. Molit..

[11] Stejskal V., Bostlova M., Nesvorna M., Volek V., Dolezal V., Hubert J. (2017). Comparison of the resistance of mono and mul-tilayer packaging films to stored product insects in a laboratory test. Food Control.

[12] Riudavets J., Salas I., Pons M.J. (2007). Damage characteristics produced by insect pests in packaging film. J. Stored Prod. Res..

[13] Germinara G.S., De Cristofaro A., Rotundo G. (2008). Behavioral responses of adult *Sitophilus granarius* to individual cereal volatiles. J. Chem. Ecol..

[14] Murata M., Imamura T., Miyanoshita A. (2008). Infestation and development of *Sitophilus* spp. in pouch-packaged spaghetti in Japan. J. Econ. Entomol..

[15] Trematerra P., Savoldelli S. (2014). Pasta preference and ability to penetrate through packaging of *Sitophilus zeamais* Motschulsky (Coleoptera: Dryophthoridae). J. Stored Prod. Res..

[16] Schöller M., Prozell S., Suma P., Russo A., Athanassiou C.G., Arthur F.H. (2018). Biological control of stored product insects. Recent Advances in Stored Product Protection.

[17] Hou X., Fields P., Taylor W. (2004). The effect of repellents on penetration into packaging by stored-product insects. J. Stored Prod. Res..

[18] Scheff D.S., Sehgal B., Subramanyam B. (2018). Evaluating penetration ability of *Plodia interpunctella* (Hubner) (Lepidoptera: Pyralidae) larvae into multilayer polypropylene packages. Insects.

[19] Vrabič Brodnjak U., Jordan J., Trematerra P. (2020). Resistance of packaging against infestation by *Sitophilus zeamais*. J. Food Sci. Technol..

[20] Vrabič Brodnjak U., Jordan J., Trematerra P. Durable pasta packaging with bio-based barrier to prevent insect infestation. Proceedings of the 11th International Conference on Simulation and Modelling in the Food and Bio-Industry, FOODSIM.

[21] Germinara G.S., Conte A., Lecce L., Di Palma A., Del Nobile M.A. (2010). Propionic acid in bio-based packaging to prevent *Sitophilus granarius* (L.) (Coleoptera, Dryophthoridae) infestation in cereal products. Innov. Food Sci. Emerg. Technol..

[22] Mullen M.A., Vardeman J.M., Bagwell J., Hagstrum D.W., Phillips T.W., Cuperus G. (2012). Insect Resistant Packaging. Stored Product Protection.

[23] Heeps J. (2006). Insect Management for Food Storage and Processing.

[24] Riudavets J., Pons M.J., Messeguer J., Gabarra R. (2018). Effect of CO_2_ modified atmosphere packaging on aflatoxin production in maize infested with *Sitophilus zeamais*. J. Stored Prod. Res..

[25] Fields P., Xie Y., Hou X. (2001). Repellent effect of pea (*Pisum sativum*) fractions against stored-product insects. J. Stored Prod. Res..

[26] Fields P. (2006). Effect of *Pisum sativum* fractions on the mortality and progeny production of nine stored-grain beetles. J. Stored Prod. Res..

[27] CABI *Compendium*: Status as Determined by CABI Editor. https://www.cabi.org/isc/datasheet/10850.

[28] Harborne J.B., Boulter D., Turner B.L. (1971). Chemotaxonomy of the Leguminosae.

[29] Bell E.A., Harborne J.B. (1978). Toxins in seeds. Biochemical Aspects of Plant and Animal Coevolution.

[30] Coombs C.W., Billings C.J., Porter J.E. (1977). The effect of yellow split-peas (*Pisum sativum* L.) and other pulses on the productivity of certain strains of *Sitophilus oryzae* (L.) (Col. Curculionidae) and the ability of other strains to breed thereon. J. Stored Prod. Res..

[31] Holloway G.J. (1986). The potency and effect of phytotoxins within yellow split-pea (*Pisum sativum*) and adzuki bean (*Vigna angularis*) on survival and reproductive potential of *Sitophilus oryzae* (L.) (Coleoptera: Curculionidae). Bull. Ent. Res..

[32] Bodnaryk R., Fields P.G., Xie Y., Fulcher K. (1999). Insecticidal Factors from Field Pea. U.S. Patent.

[33] Delobel B., Grenier A., Gueguen J., Ferrasson E., Mbailao M. (1998). Utilisation d’un Polypeptide Dérivé d’une Albumine PA1b de légumineuse Comme Insecticide. French Patent.

[34] Beleggia R., Platani C., Spano G., Monteleone M., Cattivelli L. (2009). Metabolic profiling and analysis of volatile composition of durum wheat semolina and pasta. J. Cereal Sci..

[35] Germinara G.S., Beleggia R., Fragasso M., Pistillo M.O., De Vita P. (2018). Kernel volatiles of some pigmented wheats do not elicit a preferential orientation in *Sitophilus granarius* adults. J. Pest. Sci..

[36] Vandendool H., Kratz P.D. (1963). A generalization of the retention index system including linear temperature programmed gas-liquid partition chromatography. J. Chromatogr. A.

[37] Sokal R.R., Rohlf F.J. (1995). Biometry: The Principles and Practice of Statistics in Biological Research.

[38] Najar-Rodriguez A.J., Galizia C.G., Stierle J., Dorn S. (2010). Behavioral and neurophysiological responses of an insect to changing ratios of constituents in host plant-derived volatile mixtures. J. Exp. Biol..

[39] Webster B., Gezan S., Bruce T., Hardie J., Pickett J. (2010). Between plant and diurnal variation in quantities and ratios of volatile compounds emitted by *Vicia faba* plants. Phytochemistry.

[40] Cha D.H., Linn C.E., Teal P.E.A., Zhang A., Roelofs W.L., Loeb G.M. (2011). Eavesdropping on plant volatiles by a specialist moth: Significance of ratio and concentration. PLoS ONE.

[41] Bruce T.J., Wadhams L.J., Woodcock C.M. (2005). Insect host location: A volatile situation. Trends Plant Sci..

[42] Bruce T.J., Pickett J.A. (2011). Perception of plant volatile blends by herbivorous insects-finding the right mix. Phytochemistry.

[43] Germinara G.S., Rotundo G., De Cristofaro A., Giacometti R. (2002). Risposte elettroantennografiche di *Sitophilus granarius* (L.) e *S. zeamais* Motschulsky a sostanze volatili dei cereali. Tec. Molit..

[44] Feussner I., Wasternack C. (2002). The lipoxygenase pathway. Ann. Rev. Plant. Biol..

[45] Matsui K. (2006). Green leaf volatiles: Hydroperoxide lyase pathway of oxylipin metabolism. Curr. Opin. Plant Biol..

[46] Hubert J., Munzbergova Z., Santino A. (2008). Plant volatile aldehydes as natural insecticides against stored-product beetles. Pest Manag. Sci..

[47] Azarnia S., Boye J.I. (2011). Flavour Compounds in Legumes: Chemical and Sensory Aspects. Progress in Food Science and Technology.

[48] Khrisanapant P., Kebede B., Leong S.Y., Oey I. (2019). A Comprehensive Characterisation of Volatile and Fatty Acid Profiles of Legume Seeds. Foods.

[49] Pasqualone A., Paradiso V.M., Summo C., Caponio F., Gomes T. (2014). Influence of Drying Conditions on Volatile Compounds of Pasta. Food Bioprocess Technol..

[50] Germinara G.S., Conte A., De Cristofaro A., Lecce L., Di Palma A., Rotundo G., Del Nobile M.A. (2011). Electrophysiological and behavioural activity of (*E*)-2-hexenal in the granary weevil and its application in food packaging. J. Food Prot..

[51] Pretheep-Kumar P., Mohan S., Ramaraju K. (2004). Protein-enriched pea flour extract protects stored milled rice against the rice weevil, *Sitophilus oryzae*. J. Insect Sci..

[52] Gressent F., Rahioui I., Rahbé Y. (2003). Characterization of a high-affinity binding site for the pea albumin 1b entomotoxin in the weevil *Sitophilus*. Eur. J. Biochem..

